# Employee cooperative behavior in organizations: a vignette experiment on the relationship between training and helping intentions

**DOI:** 10.1111/ijtd.12128

**Published:** 2018-07-27

**Authors:** Nikki van Gerwen, Vincent Buskens, Tanja van der Lippe

**Affiliations:** ^1^ Department of Sociology/ICS Utrecht University Utrecht The Netherlands

## Abstract

The mutual‐investment model predicts a positive relation between investments in training and employees’ willingness to behave cooperatively. In this paper, we argue that the extent to which employees increase their cooperative behavior after receiving training depends on the type of training provided, the skillfulness of the employee and the cohesiveness of the team. Focusing on intentions to help coworkers as an indicator for cooperative behavior, we conducted a vignette experiment among 2388 employees working in 127 organizations from four European countries. Multilevel analyses show that training increases employees’ intentions to help coworkers. Training promotes helping intentions the most when organizations provide general instead of firm‐specific training and when given to employees with limited skills. Whereas employees in cohesive teams indicate higher intentions to help coworkers than employees in non‐cohesive teams, training promotes helping intentions equally in both types of teams.

## Introduction

Investments in training are an important determinant for organizational performance. By participating in training, employees maintain and update their skills and knowledge enhancing their own and the organization’s productivity (Bassanini et al., 2005; Evans & Davis, 2005; Hillmert et al., 2017; Huselid, 1995; Vogtenhuber, 2015). The extent to which investments in training have these intended effects is argued to be dependent on employees’ willingness to behave cooperatively (Appelbaum et al., 2000; Sun et al., 2007). Employee cooperative behavior consists of voluntary actions that are highly valuable to the organization, but costly to the individual employee as they are not directly recognized by formal reward systems (Koys, 2001; Pierce & Maurer, 2009). Yet, there is little research focusing on whether and under which conditions training can promote employee cooperative behavior. This paper aims to fill this void by assessing the role of training as a possible determinant for employee cooperative behavior.

Applications of social exchange theories, such as the mutual‐investment model, are often used to explain why employees engage in cooperative behavior (Blau, 1963; Tsui et al., 1997). The basic premise of the mutual‐investment model is that employers and employees are embedded in exchange relationships and are willing to invest in order to maintain a balanced relationship. Investments from the employer, such as training, potentially distort the balance of the exchange relationship. To restore the balance employees are willing to increase their cooperative behavior. In this paper, we take the mutual‐investment model as starting point and argue that the balance of the exchange relationship is affected differently depending on the type of training provided, the skills and knowledge of the employee and the cohesiveness of the team. We focus on these characteristics as employers often take differences in terms of skills and knowledge into account when deciding the type of training to provide to which employee. Team cohesion is key because cooperative behavior is more likely to occur in groups characterized by social norms encouraging employees to cooperate (Cabrera & Cabrera, 2005; Shen et al., 2013). For organizations, it is important to understand the conditions under which training can be expected to increase employee cooperative behavior as this often is what guides their human capital investment decisions. We pose the following research question: ‘To what extent and under which conditions can investments in training increase employees’ intentions to behave cooperatively?’ In particular: ‘How does the effect of training on employees’ intentions to behave cooperatively depend on the type of training provided, the skillfulness of the employee receiving training, and the cohesiveness of the employee’s team?’

Previous research has mainly relied on survey data for understanding when employees cooperate (Podsakoff et al., 2000). Although survey data provide valuable insights for understanding the relation between training and cooperative behavior, they are less appropriate for investigating causal relations. The non‐random selection of employees into training makes it difficult to distinguish the causal effect of training from other unobservable factors that drive training participation as well as cooperative behavior. It might be just as likely that cooperative behavior is an antecedent of training, rather than training being the antecedent of cooperative behavior (Podsakoff et al., 2003). Vignette experiments are well‐suited for investigating causal relations as variations in training participation can be exogenously determined and systematically varied (Podsakoff et al., 2003; Wallander, 2009). Due to this advantage, we employ a vignette study, or a factorial survey design, to answer our research questions. The main disadvantage of this strategy is that we are measuring the effects of training on employees’ intentions to cooperate rather than their actual behavior. As intentions to behave cooperatively are generally seen as a good proxy for actual cooperative behavior (for example: Cho & Lewis, 2012), we do not perceive this to be too problematic. By employing a vignette study, we contribute to the call for more experimental research in understanding the antecedents of cooperative behavior (Podsakoff et al., 2000).

In this paper, we focus on one specific type of cooperative behavior: intentions to help coworkers. We focus on intentions to help because the shift toward smaller and more flexible teams has stressed the importance of knowledge sharing among employees in the functioning of the organization (Frenkel & Sanders, 2007; Wang & Noe, 2010). However, voluntarily sharing knowledge by helping coworkers is costly for individual employees. By helping coworkers, employees are giving up time they would have spent on their own working tasks otherwise. Employees have to make up for this time by, for example, working overtime. Therefore, employees have an incentive to refrain from behaving cooperatively and not help their coworkers in order to minimize individual effort (Koys, 2001). These situations are also referred to as *cooperation problems* (Olson, 1965; Ostrom, 2000).

Summarizing, this paper contributes to the literature examining the determinants of employee cooperative behavior in two ways. First, although there are studies pointing toward training as a possible incentive to motivate employees to cooperate, few have zoomed in on training and the conditions under which training can be expected to have these effects. We fill this void by taking the mutual‐investment model as a starting point and incorporating the effects of training and additional variables closely linked to training into the model. To our knowledge, we are among the first to extend the mutual‐investment model in this way. The characteristics we take into account are to some extent open to organizational influence making the results of this paper informative for organizations regarding how to promote cooperative behavior. A second contribution is the implementation of a vignette experiment allowing us to sustain causal inferences more convincingly compared to when cross‐sectional data are used. Lastly, in contrast to previous research that has mainly relied on data from one sector within one country, our vignette experiment was conducted in six different sectors within four European countries allowing for a more robust test of the relation between training and employees’ intentions to behave cooperatively.

## Theoretical framework

In applying the mutual‐investment model, we make two assumptions. First, we assume that employees and employers are motivated to balance the exchange relationship to maintain the relationship. Employers rely on their employees for organizational success and employees rely on their employers for, among other things, income. Therefore, both actors have an incentive to maintain the relationship. Second, we assume that training is valued positively by employees. We specifically look at situations in which employees want to participate in training and exclude situations in which participating in training is obligatory, for example, because an employee is new to the organization. In doing so, we distinguish investments in training that can be seen as a burden for employees from investments in training that are more likely to be interpreted as the employer doing something extra for her employee.^1^

### The effect of training and the type of training

Combining insights from several social‐exchange theories, such as leader–member exchange models (Wayne et al., 1997), gift exchange models (Akerlof & Yellen, 1990) and models based on a psychological contract between employers and employees (Rousseau, 1995), the mutual‐investment model (Tsui et al., 1997) is often put forward to understand why and when employees can be motivated to engage in cooperative behaviors. The basic premise of this model is that both parties are willing to invest some of their own resources in order to maintain a balanced relationship (Tsui et al., 1997). By providing training, the employer offers time and money for the employee to become more productive and to be prepared for higher ranking positions. Relationally, she signals that she expects to have a long‐term relationship with this employee (Lindenberg, 2000). This investment temporarily unbalances the exchange relationship. As a result, the employee feels that he is receiving more than he is giving. In order to restore the balance, the employee is expected to reciprocate to the training by increasing his efforts, for example by behaving cooperatively. Alternatively, employees not receiving training might feel that they are receiving less than they are giving and develop feelings of frustration or relative deprivation. Research has suggested that such employees tended to have lower intentions to engage in cooperative behaviors (Bolino & Turnley, 2009).

Gouldner’s (1960) reciprocity principle thus resides ‘at the core of the exchange concept’ (Blau, 19633, p. 140). Employers exchange investments in training for cooperative behavior of their employees. These cooperative behaviors can be directed toward the organization or toward coworkers. When they are directed toward the organization, we speak of direct reciprocity (i.e. ‘you help me, I help you’), whereas cooperative behavior directed toward coworkers are often explained by generalized reciprocity (i.e. ‘I help you and you help someone else’) (Baker & Bulkley, 2014; Hyde, 2000). The generalized reciprocity principle produces greater knowledge sharing and forms of assistance, which would otherwise not occur (Ipe, 2003; Kollock, 1994). Clearly, this behavior is also beneficial to the organization. In line with previous research and in line with the mutual‐investment model (Ipe, 2003; Kampkötter & Marggraf, 2015; Kollock, 1994; Lambooij et al., 2007; Tsui et al., 1997), we formulate the first hypothesis:

Hypothesis 1: Employees receiving training indicate more helping intentions than employees not receiving training.

The main objective of training is for employees to learn new skills or to keep their current skills updated. As outlined above, employees are expected to reciprocate to this signal by increasing their cooperative behavior. The extent to which training distorts the balance of the exchange relationship might differ depending on the type of skills acquired in training. Becker (1975) distinguishes general from firm‐specific training. In general training, employees acquire skills and knowledge that can be applied both in their current organization as well as in other organizations, whereas firm‐specific training enhances skills and knowledge specific to the organization for which an employee is currently working. When general training is provided, employees are trained to be flexible and multi‐skilled and become attractive to other employers which benefits their mobility (Martin, 2007). Firm‐specific training has no or even a negative effect on employees’ mobility, as this type of training only increases the value of an employee to his current organization (Loewenstein & Spletzer, 1999). When both types of training are offered by the organization, this difference makes the employees perceive general training as a larger investment from the employer than firm‐specific training.

Research showing that employees demonstrate higher levels of turnover intentions when general training is provided than when firm‐specific training is provided (Benson, 2006) underlines that employees realize that general training increases their labor market position and is more valuable than firm‐specific training. In terms of the exchange relationship, this difference will affect the balance of the exchange relationship differently. General training is more valuable for the employee and can therefore be expected to distort the exchange relationship more than firm‐specific training. To restore the balance, employees receiving general training are willing to exert more effort than employees receiving firm‐specific training. We pose the following hypothesis:

Hypothesis 2: The positive effect of receiving training on employees’ helping intentions is larger when general training is provided, compared to when firm‐specific training is provided.

### The moderating role of skills and knowledge

Thus far, we have neglected the idea that employers select specific employees for training based on their skills and knowledge. According to Tanova and Nadiri (2005), such training selection methods are an important aspect of the organizational strategy. Employers train specific groups of employees because they expect different results from the investment. However, before we turn to the moderating role of skills and knowledge, it is important to recognize that employee differences in terms of skills and knowledge might directly affect their intentions to help others.

Employees’ decisions to help are not solely based on their willingness, but are at least partly determined by their ability to do so. Employees with extensive skills and knowledge have more resources and are often better able to help their coworkers than employees with limited skills and knowledge. In other words, there exists inequality among employees in terms of ability to help others. Due to this inequality, the costs of helping are lower for employees with extensive skills and knowledge than for employees with limited skills and knowledge. When considering that employees are not only embedded in an exchange relationship with their employer, but are additionally embedded in exchange relationships with coworkers, decisions to help might emerge from a sense of social obligation (Blau, 1963; Mossholder et al., 2011). Employees with extensive skills and knowledge might realize that they are better suited to help than their coworkers with limited skills and knowledge and therefore feel more obliged to do so. This argument is in line with the notion of noblesse oblige (Homans, 1961), which implies that employees with extensive skills and knowledge feel it is their responsibility to help (see also: De Cremer & Van Dijk, 2009). Please note that this does not imply that employees with limited skills and knowledge never help their coworkers. We formulate the following hypothesis:

Hypothesis 3: Employees with extensive skills and knowledge indicate more helping intentions than employees with limited skills and knowledge.

When employees with extensive skills and knowledge receive training, their resources will increase even further. As a result, the inequality between employees with extensive skills and employees with limited skills becomes larger making it less costly for the employee who received training to help coworkers. At the same time, the investment in training has unbalanced the exchange relationship between the employer and the employee. Consequently, employees with extensive skills and knowledge receiving training feel even more obliged to help their coworkers. The perceived obligation to help is fostered not only because the inequality between themselves and their coworkers has grown, but also because the investment in training has unbalanced the exchange relationship with the employer. Employees with extensive skills and knowledge can thus solve two problems at once by behaving cooperatively. Contrastingly, the inequality between employees in terms of ability to help others decreases when employees with limited skills and knowledge receive training, and it remains less self‐evident to what extent the employees with limited skills can help coworkers. We formulate the following hypothesis:

Hypothesis 4: The positive effect of training on employees’ helping intentions is larger for employees with extensive skills and knowledge, compared to employees with limited skills and knowledge.

### The moderating role of cohesiveness

In the previous section, we argued that employees are not only embedded in an exchange relationship with their employer, but are also embedded in exchange relationships among themselves. Next, we consider that the nature of these exchange relationship among coworkers in terms of cohesion can motivate employees to cooperate. Coleman (1988, 1990 ) was among the first to stress the positive effect of cohesion on cooperative behavior. Cohesion refers to affinity between employees and their identification as a group. Cohesive teams promote cooperative behavior in two ways: by facilitating trust between coworkers and by reducing the risks of opportunistic behavior via sanctioning mechanisms.

Members of highly cohesive groups share a strong social identity which increases their willingness to help other members of the group. This is often attributed to the level of trust within the group (Cabrera & Cabrera, 2005; Frenkel & Sanders, 2007; Shen et al., 2013). Cohesive ties to coworkers amplify the pressure to reciprocate past cooperative behavior (Gargiulo & Benassi, 2000). Employees who received help from coworkers in the past are expected to reciprocate and help coworkers themselves. This also implies that employees helping coworkers can trust the favor will be returned in the future. The amplified reciprocity norm in cohesive groups reduces the uncertainty of the exchange and motivates employees to behave cooperatively (see also: Rooks et al., 2000).

Simultaneously, members of cohesive groups are more likely to sanction opportunistic behavior from one of their group members than members of non‐cohesive groups. An employee not returning the favor to a coworker who has helped him in the past is more likely to be told off by coworkers in cohesive groups. Such sanctioning mechanisms reduce the risks of opportunistic behavior and facilitate trust between coworkers, which subsequently enhances employees’ willingness to behave cooperatively (see also Raub & Weesie, 1990). Employees linked through coworkers are more likely to conform to norms of reciprocity as failure to reciprocate past behavior may result in sanctions from coworkers. Previous research also shows a positive association between social cohesion and employee cooperative behavior (Frenkel & Sanders, 2007; Ng & van Dyne, 2005; Shen et al., 2013). We pose the following hypothesis:

Hypothesis 5: Employees embedded in more cohesive teams indicate more helping intentions than employees embedded in less cohesive teams.

The mutual‐investment model predicts that employees are more willing to help coworkers after receiving training. At the same time, employees in cohesive teams are expected to be more willing to help coworkers because employees within such teams can trust each other to reciprocate past cooperative behavior. However, employees face a limit in the extent to which they can behave cooperatively. When considering that employees in cohesive teams are already behaving more cooperatively than employees in non‐cohesive teams (Frenkel & Sanders, 2007; Ng & van Dyne, 2005; Shen et al., 2013), this implies that employees in cohesive teams have less to give in terms of cooperative behavior than employees in non‐cohesive teams. Or to put it differently, employees in cohesive teams are facing a ‘ceiling effect’ limiting the extent to which training can promote cooperative behavior within such teams. Employees in non‐cohesive teams are less motivated to help coworkers unless training is provided and are able to increase their cooperative behavior more. As such, the effects of training and cohesion can be seen as substitutes of each other. Kong and colleagues (2017) tested this mechanism focusing on voice behavior as an indicator for cooperative behavior. They conclude that team cohesion promotes cooperative behavior more among employees having a low‐quality exchange relationship with their employer, compared to employees having a high‐quality exchange relationship. We formulate the following hypothesis:

Hypothesis 6: The positive effect of training on an employee’s intention to help coworkers is larger in less cohesive teams, compared to more cohesive teams.

## Methodology and data

Supplementary information about the methodology can be found in the Appendix.

### Sample

The main objective of this study is to empirically establish the relationship between training and employees’ intentions to help coworkers. To achieve this objective, we designed a vignette experiment. The experiment is part of the European Sustainable Workforce Survey (ESWS). The ESWS is a multi‐actor survey conducted within organizations in Bulgaria, Finland, Germany, Hungary, the Netherlands, Portugal, Spain, Sweden and the United Kingdom collected in 2015/2016 (van der Lippe, 2016). Organizations were selected from statistical and commercial business registers and stratified according to country, sector and size. In each country, organizations from the manufacturing, higher education, health care, telecommunication, transport and logistics and finance and banking sectors were selected. Within each sector, organizations were additionally selected on their number of employees (small: 20–99 employees, medium: 100–249 employees and large: 250 employees or more). Within each organization, three or four teams were selected together with the HR‐manager to participate in the survey. Employees were contacted at work and requested to fill out a survey, either online or on paper. In total, 11,011 employees from 259 organizations participated in the ESWS. After completing the survey, the 6134 employees from 137 organizations in Bulgaria, Germany, the Netherlands and Portugal were asked to participate in a short ‘thought experiment’, which followed immediately after and is the vignette experiment under study in this paper. The response rate for the survey was 61.4 per cent and the response rate for the vignette was 45.9 per cent, leading to 2816 respondents from 129 organizations. We exclude 428 employees who did not complete the experiment. The final sample includes 2388 employees from 129 organizations. The 2388 employees were each presented with six vignettes, bringing the total number of observations to 14,328.

### Vignettes

A vignette is a short description of a hypothetical situation in which the factors considered theoretically relevant to a decision are systematically varied in the form of a short story (Auspurg & Hinz, 2015; Rossi & Anderson, 1982). With survey data it is hard to distinguish whether employees receive training because they behave cooperatively, or whether they behave cooperatively because they received training. Moreover, given that surveys often collect data on both the independent and dependent variables from the same respondent at the same time, survey data potentially suffer from a common method bias (Podsakoff et al., 2003). Employing a vignette experiment (or factorial survey design) helps overcoming these problems. The exogenous variation in the independent variables allows for a clear distinction between cause and effect as the independent variables, such as training participation, are determined and thus observed before respondents are asked regarding their intentions to help coworkers. This allows us to sustain causal inferences regarding the effects of the hypothetical situation on intentions to help coworkers more convincingly than when survey data are used. At the same time, because the independent variables are fixed by the experimenter and observed before the respondents answer the questions regarding their helping intentions, employing a vignette experiment additionally prevents the data from suffering from a common method bias (Podsakoff et al., 2003).

Critics argue that vignette experiments lack external validity and do not accurately capture real‐world decision‐making. They argue that vignette experiments are prone to several types of biases, such as hypothetical decision bias and social desirability bias (Krosnick et al., 2014). Hainmueller and colleagues (2015) compared results from vignette experiments with a behavioral benchmark of voting in naturalization referendums and suggest that in order to maximize external validity: ‘the sample needs to be carefully chosen to match the target population’ and ‘the experimental design should be carefully crafted to mimic the incentives that respondents would face making the decision in the real world’. Therefore, we choose to set out the experiment among actual employees and choose a hypothetical situation that closely relates to situations that employees may encounter in their job. By doing this, we aim to enhance the external validity of our findings compared to studies using student populations or online recruitment systems, while simultaneously benefiting from the increase in internal validity which accompanies the experimental nature of vignette studies.

**Figure 1:**
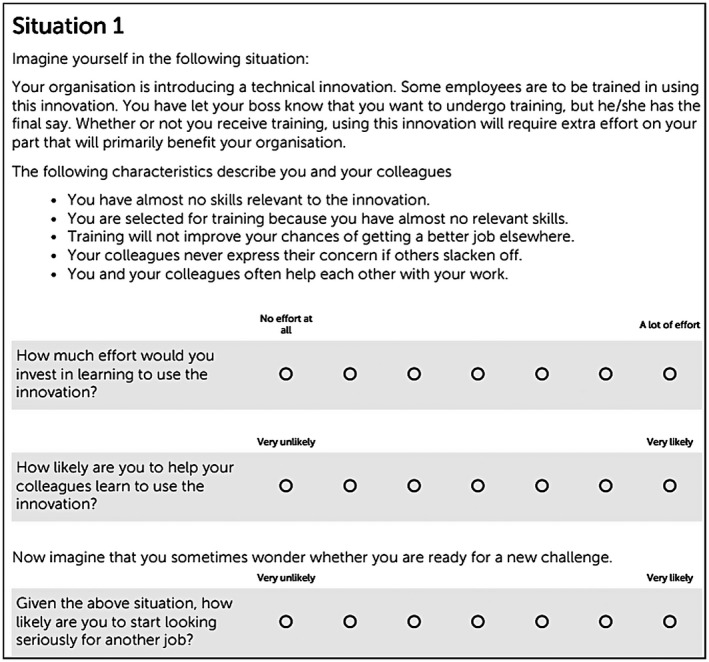
: Example of a vignette as presented to respondents

Respondents participating in the experiment were given six hypothetical situations (the vignettes) describing a fictional situation occurring within their current organization. In this situation, the organization is introducing a technical innovation. Since the experiment is set out in different sectors, we do not specify the exact nature of the technical innovation. We describe that some respondents are to be trained in using this innovation and state that all have notified their boss that they want to participate in training. This last statement encourages respondents to think positively of the training. Respondents were asked to indicate the likelihood that they would help their coworkers learn to use the technical innovation on a seven‐point Likert scale ranging from very unlikely to very likely (see Figure 1).

The six vignettes given to each respondent were selected from the pool of vignettes generated by varying the five factors (‘dimensions’) which we consider likely to affect employees’ intentions to help coworkers: (1) their skills and knowledge; (2) their training participation; (3) the type of training provided; (4) the existence of social norms toward helping coworkers; and (5) the existence of sanctioning mechanisms. For each of these dimensions, two categories (‘levels’) were defined. Table 1 summarizes these dimensions and their corresponding levels. Before presenting the vignettes, a short instruction was given to the respondents, explaining that they were expected to imagine themselves in the situations described on the vignettes and asking them to answer the questions as if these situations occurred to them in their current job.

**Table 1:** : Dimensions and categories used in the scenarios

	Dimension	Level	Text
1	Skills and knowledge	0	You have almost no skills relevant to the innovation
		1	You have quite some skills relevant to the innovation
2	Training^a^	0	You are not selected for training.
		1	You are selected for training
3	General training^b^	0	Training will not improve your chances of getting a better job elsewhere
		1	Training will improve your chances of getting a better job elsewhere
4	Social norms	0	You and your colleagues never help each other with your work
		1	You and your colleagues often help each other with your work
5	Sanction	0	Your colleagues never express their concern if others slacken off
		1	Your colleagues usually express their concern if others slacken off

a Please note that for two‐thirds of the vignettes the text for training dimension was extended with either the phrase ‘because you have almost no skills relevant to the innovation’ or the phrase ‘because you have quite some skills relevant to the innovation’ depending on the value for the dimension skills and knowledge on the vignettes. This distinction did not make any difference for the tests of our hypotheses and we neglect these extended formulations here.

b Please note that in the case of no training the vignette would read: training *would not* or *would* improve your chances of getting a better job elsewhere instead of *will not* or *will*.

The full set of all possible vignette combinations contains (2*2*2*2*2=) 32 different vignettes. In order to ensure enough within‐person variation on the key dimension training, we adopted a stratified sampling approach to create the subsets: within each subset of six vignettes, we imposed three of the vignettes to contain training and three of the vignettes not to contain training. Other than this imposed variation in training participation, all values for the remaining variables were randomly selected. All 32 vignettes were equally likely to occur in the subset of six vignettes given to each respondent. Vignettes were presented to the respondents in a random order. All vignettes were presented in the format of Figure 1, the only thing that changed between the vignettes was the independent variables which were on the vignette described by the bullet points under the header ‘The following characteristics describe you and your colleagues’. Once employees answered a specific vignette, they continued to the next vignette until respondents rated all six vignettes. The vignettes were designed in English and presented to the respondents in their national language. Translations were double‐checked using the back‐translation method. Participating in the experiment took respondents on average 12 minutes.

### Construction of variables

The dependent variable *intention to help coworkers* is measured by the question: ‘How likely are you to help your coworkers to use this innovation?’ Answer categories ranged from 0 (very unlikely) to 6 (very likely). We focus specifically on this question and not on the other two questions asked on the vignettes as we feel that this question captures employees’ intentions to behave cooperatively most explicitly. Willingness to learn and intentions to leave might be influenced by separate individual characteristics such as ambitions to develop in other directions and are therefore less well interpretable as direct measures of cooperative behavior.

The independent variables are constructed as dummy variables and relate directly to the vignette characteristics as presented in Table 1. As outlined in the theory section, previous research has identified two ways in which cohesion can foster cooperative behavior: cooperative behavior is more likely to occur in highly cohesive groups because *social norms* toward helping each other are more likely to exist and because members of cohesive groups are more likely to *sanction* each other for deviating from this norm. Consequently, we use two proxies for cohesion: the existence of social norms and the existence of sanctioning mechanisms.

It could be argued that in some sectors cooperative behavior occurs more frequently than in others: for example, employees in the health care sector work together more frequently which might affect their intentions. To control for this and similar effects, we add sector dummies to the model. To control for similar differences between countries, we add country dummies to our model.

### Analytical strategy

A key characteristic of a factorial survey design is that the vignette, and not the respondent, is the unit of analysis (Hox, 2010). Each respondent was asked to answer six vignettes; therefore, the vignettes are nested within the employees. The appropriate way to analyze such nested data is by means of multilevel regression analysis. The analytical strategy is as follows: first, we estimate an intercept only (or empty) model, which includes the dependent variable and the respondent and vignette‐level variance. Since the data contain multiple employees from the same organization, we also add an organizational level to the model. The Intra Class Correlation (ICC) is estimated by dividing the level variance by the total variance. In Model 1, we add the independent variables to our model to determine whether the variance of the different levels can be explained by these specific predictors. We additionally add dummy variables to control for possible sector and country differences to this model. In Model 2, we add the interaction terms. The underlying multivariate multilevel model has three levels: the vignette level (*N *= 14,328), the respondent level (*N *= 2388) and the organization level (*N *= 127).

## Results

### Descriptive statistics

Correlations and descriptions of the dependent and independent variables are displayed in Table 2. Over all vignettes respondents scored on average 4.2 (std = 1.655) on a scale from 0 to 6 regarding their intentions to help coworkers. Table 2 shows the correlations between the dependent and the independent variables. Except for the correlations between the training variables, all correlations between the independent variables are weak reflecting the factorial design. The vignette describing an employee with extensive skills and knowledge in combination with a general training seems to occur slightly more often in our sample (note this includes both scenarios in which training is provided and scenarios in which training is not provided).

**Table 2:** : Correlations and descriptive statistics

	Mean	Std.	1	2	3	4	5
1. Intentions to help	4.207	1.655					
2. Training	0.500	0.500	0.126***				
3. Skills and knowledge	0.497	0.500	0.080***	0.005			
4. General training	0.506	0.500	0.042***	0.002	0.019*		
5. Social norms	0.506	0.500	0.139***	−0.012	−0.004	−0.001	
6. Sanction	0.505	0.500	0.044***	0.000	−0.001	−0.009	0.012

Note:

*N *= 14,328.

*p < 0.05,**p < 0.01,***p < 0.001 (two‐sided tests).

### Explanatory results

Model 1 in Table 3 presents the multilevel model estimated to test hypotheses regarding employees’ intentions to help. The ICC is calculated in an empty model (results available upon request). Roughly 44 per cent of the total variance is represented at the vignette level. The remaining variance is mainly represented at the respondent level (52 per cent). This indicates that most of the variance resides between and within respondents and only a small part of the variance is due to employees working in different organizations (4 per cent).

**Table 3:** : Multivariate multilevel analyses to explain intentions to help: 14,328 vignettes in 2388 respondents nested in 127 organizations, robust standard errors

		Model 1		Model 2	
		Coefficient	*S.E*.	Coefficient	*S.E*.
**Fixed part**					
Constant		3.268***	0.094	3.238***	0.100
*Independent variables*					
Training		0.420***	0.024	0.478***	0.056
Skills and knowledge		0.276***	0.024	0.349***	0.036
Social norms		0.452***	0.031	0.441***	0.037
Sanction		0.113***	0.024	0.128***	0.034
General training		0.126***	0.021	0.108***	0.030
Training*general training				0.034	0.041
Training*skills				−0.146**	0.044
Training*social norms				0.022	0.038
Training*sanction				−0.023	0.044
*Control variables*					
Sector (ref = Manufacturing)					
	Health care	−0.088	0.118	−0.087	0.118
	Higher education	−0.110	0.102	−0.109	0.102
	Transport	−0.051	0.140	−0.050	0.140
	Financial services	0.019	0.105	0.020	0.105
	Telecommunication	0.014	0.134	0.015	0.134
Country (ref = Netherlands)					
	Germany	0.114	0.129	0.114	0.129
	Portugal	0.601***	0.089	0.600***	0.088
	Bulgaria	0.386***	0.083	0.385***	0.083
**Random part**					
Variance (vignette level)		1.180	0.047	1.178	0.046
Variance (respondent level)		1.326	0.074	1.327	0.074
Variance (organization level)		0.050	0.015	0.050	0.016
**Fit statistics**		Parameter	DF	Parameter	DF
Deviance		47976.738	17	47959.906	21
Difference in deviance				16.832	4

*p < 0.05,**p < 0.01,p ***< 0.001 (two‐sided tests).

In Model 1, we add the effects for the vignette predictors while simultaneously controlling for sector and country differences. We see that training significantly increases employees’ intentions to help coworkers, confirming Hypothesis 1. The results also illustrate that employees with extensive skills and knowledge demonstrate significantly higher levels of intentions to help coworkers than employees with limited skills and knowledge. Therefore, we are also able to confirm Hypothesis 3. Results from Model 1 additionally show evidence to confirm Hypothesis 5: the existence of social norms toward helping colleagues and the existence of sanctioning mechanisms enhance employees’ intentions to help coworkers. Finally, Model 1 suggests that general training increases helping intentions more than firm‐specific training, but in this model, we cannot distinguish to what extent this relates to employees who do or do not receive training.

Results from Model 1 show no sector differences in relation to employees’ intentions to help coworkers. Results indicate the existence of country differences: employees from the Netherlands indicate significantly less helping intentions than employees from Portugal and Bulgaria.^2^

In Model 2, we add the interaction terms. The results show a non‐significant positive effect for the interaction between training and the type of training. This implies that the main effect of general training can be interpreted as follows: employees who receive training indicate significantly more helping intentions when they receive general training than when they receive firm‐specific training, but an almost equally large increase in helping intentions holds for employees who do not receive training (in case this training would have been general training). In other words, providing general training increases helping intentions in general more than providing firm‐specific training, regardless of whether employees themselves receive the training or not. When restricting our analysis to the vignettes in which employees receive training, results show a positive effect of receiving general rather than firm‐specific training (*b* = 0.129, *p *< 0.001, results available upon request). Therefore, we can confirm Hypothesis 2: the positive effect of training on intentions to help coworkers is larger when the training provided is general rather than firm‐specific. Regarding the skillfulness of the employee to whom training was provided, results show that the effect of training on employees’ intentions to help coworkers is smaller for employees who have extensive skills and knowledge compared to employees with limited skills and knowledge. Therefore, we reject Hypothesis 4. The results show no evidence to confirm Hypothesis 6: the effect of training is not significantly stronger in non‐cohesive compared to cohesive organizations. The existence of social norms and the existence of sanctioning mechanisms do not significantly moderate the effect of training on helping intentions.

Model 2 fits the data significantly better than Model 1 (difference in deviance test: *χ* ^2^(4) = 16.832, *p *< 0.01). This means that some interaction terms are important for understanding what motivates employees to cooperate.

### Sensitivity analyses

Table 3 above illustrates country differences. In order to examine whether our results are mainly driven by one country, we ran Model 1 separately for each country. The results showed that almost all the effects found in Model 1 were found in each of the countries separately. Only the existence of sanctioning mechanisms had no significant effect on the helping intentions of Portuguese respondents. This indicates that the results found in this paper are robust over different countries. Results can be found in the supplementary material.

As employees voluntarily participated in the vignette experiment, we investigated the presence of a selection bias by predicting the probability that an employee from Bulgaria, Germany, the Netherlands and Portugal would participate in the vignette experiment based on their age, gender, level of education, number of years working for the organization and self‐reported cooperative behavior toward coworkers. Results showed the existence of a selection bias regarding age, education and self‐reported cooperative behavior. To investigate whether this bias affected the way respondents interpreted the vignette characteristics, we examined whether the effects of the vignette characteristics differed depending on age, education and self‐reported cooperative behavior. Results showed that the presence of more older employees in our sample has not affected our results. If anything, this bias has led to an underestimation of the effect of cohesiveness on intentions to behave cooperatively. The effects of training, skills and knowledge and cohesiveness were found to be stronger for higher educated employees than for lower educated employees. Given that the former were more likely to participate in our study, this implies a possible overestimation of the results. However, the effects were so small that we do not consider this to be problematic. The only selection bias that we consider playing an important part in the results of this paper is respondents’ self‐reported cooperative behavior. The effect of sanctioning mechanisms was found to be stronger for highly cooperative employees, who were more likely to participate in our study. This means that the results regarding the existence of sanctioning mechanisms may be an overestimation. Results can be found in the supplementary material.

## Discussion and conclusion

In this paper, we have examined the causal relation between investments in training and employees’ intentions to help coworkers. In doing so, we have made three important contributions. Our first contribution is theoretical. Although the mutual‐investment model has often been proposed as a theoretical model for understanding why employees behave cooperatively (e.g. Frenkel & Sanders, 2007; Lambooij et al., 2007; Mossholder et al., 2011), its boundary conditions are rarely addressed. In this paper, we started from this model and have added to the literature by investigating how the balance of the exchange relationship that is the core of the mutual‐investment model is influenced by the type of training provided, the skillfulness of the employee and the cohesiveness of the team. In doing so, we have extended the mutual‐investment model and provided a more detailed picture of the conditions under which training can be expected to increase employee cooperative behavior. Our second and third contributions are of methodological grounds. We responded to the call for more experimental research examining the antecedents of employee cooperative behavior by collecting data through a vignette experiment (Podsakoff et al., 2003), thereby showing that letting employees imagine themselves to receive training causes employees to increase their intentions to behave cooperatively. Thirdly, in contrast to previous research, we examined the relation between training and employees’ intentions to help coworkers in multiple sectors and countries increasing the validity of our findings.

Given our results, what can we conclude from this study? Can training foster employees’ intentions to behave cooperatively? Yes, training promotes employees’ intentions to help coworkers. In line with the mutual‐investment model (Blau, 1963; Homans, 1961; Tsui et al., 1997) and the reciprocity principle (Gouldner, 1960), we conclude that training temporarily unbalances the exchange relationship between an employer and her employees causing employees to enhance their intentions to help coworkers in order to restore the balance.

We have extended the mutual‐investment model by examining how the balance of the exchange relationship is affected by training dependent on three characteristics: the type of training provided, the skillfulness of the employee and the cohesiveness of the team. We conclude that the balance of the exchange relationship was affected more when general as opposed to firm‐specific training was provided. Providing general training promoted employees’ intentions to help coworkers more than providing firm‐specific training did. This is highly interesting in light of previous research illustrating that employees were more likely to leave the organization when general training is provided (Benson, 2006). Results of this paper imply a paradoxical situation for employers: even though employees may be more likely to leave the organization after receiving general training, employees also become more willing to help their coworkers.

Although we expected training to have a larger impact on the balance of the exchange relationship when provided to employees with extensive skills and knowledge than when provided to employees with limited skills and knowledge, results indicated the exact opposite. A possible explanation for this might be that employees with extensive skills and knowledge already feel obliged to help coworkers and are accordingly indicating more helping behaviors; consequently, they only have limited room left to further increase their helping behaviors after receiving training. Employees with limited skills and knowledge do not feel the obligation to help coworkers except for when training is provided. This makes that the difference in helping behavior before and after training participation is larger for employees with limited skills and knowledge than for employees with extensive skills and knowledge. For employers, this finding is interesting as it warrants them to think carefully about whom to select for training. Although selecting the higher skilled employees for training might intuitively seem to be the best strategy, this study suggests that training the employees with limited skills and knowledge potentially boosts cooperative behavior the most.

We further conclude that employees in cohesive teams were more willing to help coworkers than employees in non‐cohesive teams. This underlines the notion that cohesive ties to coworkers foster employee cooperative behavior by reducing the uncertainty of the exchange (Coleman, 1988, 1990 ). Because employees in cohesive teams were already expected to behave more cooperatively, we formulated the hypothesis that these employees have less room left for increasing their cooperative behavior after receiving training than employees working in non‐cohesive teams. However, the results did not support this. This might imply that the ‘ceiling’ that we thought to limit employees in the extent to which they could behave cooperatively does not exist. All these relations were found independent of country and sector difference increasing the robustness of our findings.

### Limitations

Although our experimental approach has several advantages compared to traditional survey designs, it also comes with some disadvantages. A possible downside of using vignette experiments is that they become too abstract. Especially because the experiment was conducted in different sectors and countries, we might wonder whether respondents found the vignette characteristics to resemble their everyday situations sufficiently enough to answer the question in accordance with their actual behavior. Another possible disadvantage of using vignette experiments is that we are restricted to measuring intentions rather than actual behavior and that we have to rely on employees imagining themselves to receive training rather than actually receiving it. Although intentions are generally seen as good proxies for actual behavior (Ajzen & Fishbein, 1977), one may speculate as to whether the results of this study can be translated into the ‘real world’. Ideally, future research should try to replicate the results of this study in a field experiment in which employees are randomly assigned training.

There are a couple of simplifications we had to make to keep our vignette experiment concise which could have influenced the generalizability of the findings. First, because the vignette experiment was set out among employees in different countries and different sectors, we had to rely on a rather vague concept of training. We did not specify the content or the length of the training; we merely specified that employees wanted to participate in the training. Previous research has illustrated that the number of training days influences employees’ reactions to training, with longer training programs being valued positively more than shorter ones (Benson, 2006; Schmidt, 2007). Similarly, we did not elaborate on the exact difference between employees with extensive skills and employees with limited skills. Especially in relation to helping coworkers, the nature of the skills might be important. One employee can have fewer skills than others. However, if the nature of the problem requires a specific skill that only the employee with fewer skills possesses, that employee is better suited to help than the employees with extensive skills. How the distribution of skills affects employees’ helping behavior is an interesting question to be addressed in future research. Also it should be kept in mind that remuneration and other reward can also be based on team performance and that this might partly explain employees’ willingness to help coworkers. Lastly, we might wonder whether employees are equally willing to help all their coworkers or whether some employees base their decision to help on the position of the person in the organization. In the current study, we did not specify who employees were helping. Future research studying whether the effects found in this paper are equally strong for employees in different positions within the organization could tease out the conditions under which employees are expected to behave cooperatively even further.

### Implications for professional practice

The results of this study have several implications for employers who seek to promote employee helping behavior. First, investments in training are likely to enhance cooperative behavior. Although previous research has illustrated that employees receiving general training are more likely to leave the organization, results of this study suggest that they are also still willing to reciprocate to the training by helping coworkers. Secondly, training might especially enhance cooperative behavior of the lower skilled employees. This is interesting as it contrasts to the more intuitive idea that higher skilled employees will benefit the most from training. Thirdly, next to investments in training, this study underlines that managers may want to consider investing in team cohesion, as this greatly influences employees’ willingness to engage in helping behavior. In other words, findings of this paper have provided suggestions for employers in why some employees respond more cooperatively to training than others. We hope these findings encourage future research that further examines the conditions under which training can be expected to increase employee cooperative behavior.

## Uncited reference

Blau (1964).

## Supporting information

Additional Supporting Information may be found online in the supporting information tab for this article.
Table A Multivariate multilevel analyses to explain intentions to help by country, robust standard errors.
Table B Logistic regression analysis predicting the probability of participation in the vignette experiment of employees (5217 employees).
Table C Multivariate multilevel analyses to explain intentions to help while controlling for selection bias.

## ENDNOTES

1 To facilitate the distinction between employers and employees, we refer to the employer with female pronouns and to the employees with male pronouns.

2 Multiple employee‐level control variables were included in the model (i.e. age, income, educational level, self‐indicated cooperative behavior, number of years the employee has been working with the current organization and whether the respondents perceived the vignette situation to be realistic or not). None of these control variables changed the results presented here and are, for reasons of parsimony, removed from the model.
