# Supplementary material for: Employee cooperative behavior in organizations: a vignette experiment on the relationship between training and helping intentions
Source: Int J Train Dev. 2018 Jul 27;22(3):192–209. doi: 10.1111/ijtd.12128 (PMC6743710; doi:10.1111/ijtd.12128)
Supplement: Supplementary file 1 — Additional Supporting Information may be found online in the supporting information tab for this article. Table A Multivariate multilevel analyses to explain intentions to help by country, robust standard errors. Table B Logistic regression analysis predicting the probability of participation in the vignette experiment of employees (5217 employees). Table C Multivariate multilevel analyses to explain intentions to help while controlling for selection bias. [file IJTD-22-192-s001.docx]

**APPENDIX: Supplementary material**

**Table A** Multivariate multilevel analyses to explain intentions to help by country, robust standard errors

|  | **The Netherlands**  3,816 vignettes in 636 respondents in 45 organizations | | **Germany**  2,280 vignettes in 380 respondents in 18 organizations | | **Portugal**  3,498 vignettes in 583 respondents in 28 organizations | | **Bulgaria**  4,734 vignettes in 789 respondents in 36 organizations | |
| --- | --- | --- | --- | --- | --- | --- | --- | --- |
| **Fixed part** | Coef. | *S.E.* | Coef. | *S.E.* | Coef. | *S.E.* | Coef. | *S.E.* |
|  |  |  |  |  |  |  |  |  |
| Constant | 3.206*** | 0.120 | 2.764*** | 0.071 | 3.940*** | 0.246 | 3.840*** | 0.150 |
|  |  |  |  |  |  |  |  |  |
| *Independent variables* |  |  |  |  |  |  |  |  |
|  |  |  |  |  |  |  |  |  |
| Training | 0.411*** | 0.035 | 0.620*** | 0.063 | 0.316*** | 0.041 | 0.412*** | 0.047 |
| Skills and knowledge | 0.337*** | 0.037 | 0.378*** | 0.069 | 0.185*** | 0.042 | 0.246*** | 0.047 |
| Social norms | 0.540*** | 0.041 | 0.632*** | 0.073 | 0.371*** | 0.060 | 0.354*** | 0.058 |
| Sanction | 0.086*** | 0.040 | 0.101*** | 0.032 | 0.030*** | 0.031 | 0.201*** | 0.043 |
| General training | 0.115*** | 0.039 | 0.204*** | 0.049 | 0.060*** | 0.032 | 0.142*** | 0.038 |
|  |  |  |  |  |  |  |  |  |
| *Control variables* |  |  |  |  |  |  |  |  |
|  |  |  |  |  |  |  |  |  |
| Sector (ref=Manufact.) |  |  |  |  |  |  |  |  |
| Health care | 0.106*** | 0.178 | 0.277*** | 0.178 | -0.018*** | 0.260 | -0.469*** | 0.195 |
| Higher education | -0.153*** | 0.129 | 0.545*** | 0.149 | 0.013*** | 0.242 | -0.398*** | 0.179 |
| Transport | -0.171*** | 0.143 | -0.322*** | 0.630 | 0.321*** | 0.327 | -0.036*** | 0.207 |
| Financial services | 0.037*** | 0.141 | 0.312*** | 0.117 | 0.116*** | 0.259 | -0.073*** | 0.171 |
| Telecommunication | -0.134*** | 0.183 | 0.269*** | 0.050 | 0.336*** | 0.344 | -0.184*** | 0.200 |
|  |  |  |  |  |  |  |  |  |
| **Random part** |  |  |  |  |  |  |  |  |
| Var (vignette) | 1.096*** | 0.057 | 1.366*** | 0.068 | 0.815*** | 0.061 | 1.412*** | 0.096 |
| Var (respondent) | 1.133*** | 0.105 | 1.152*** | 0.087 | 1.202*** | 0.077 | 1.655*** | 0.159 |
| Var (organization) | 0.014*** | 0.017 | 0.071*** | 0.036. | 0.037*** | 0.034 | 0.022*** | 0.013 |
|  |  |  |  |  |  |  |  |  |
| **Fit statistics** | Parameter | DF | Parameter | DF | Parameter | DF | Parameter | DF |
| Deviance | 12440.936 | 14 | 7862.923 | 14 | 10555.804 | 14 | 16703.663 | 14 |

**p*<0.05, ***p*<0.01, *** <0.001 (two-sided tests).

To examine the presence of a selection bias we ran a logistic regression analysis predicting the probability that an employee from Bulgaria, Germany, the Netherlands, and Portugal would participate in the vignette experiment. As predictor variables we included age, male, level of education, number of years working for the organizations, self-reported cooperative behavior and sector. Table B shows the results from the logistic regression analysis. We excluded all respondents who had a missing value on one or more of the predictor variables (*N*=917). Results show that older employees, higher educated employees, and employees scoring higher on the cooperative behavior scale were significantly more likely to participate in the vignette experiment. Gender and number of years working for the organization did not significantly affect the odds of participating.

**Table B** Logistic regression analysis predicting the probability of participation in the vignette experiment of employees (5,217 employees)

|  | Coefficient | *S.E.* |
| --- | --- | --- |
| Constant | ** 0.039*** | 0.011 |
|  |  |  |
| Age | **-1.017*** | 0.003 |
| Male | ** 1.055 | 0.060 |
| Level of education | **-1.049** | 0.020 |
| # Years working for the organization | ** 0.998 | 0.004 |
| Self-reported cooperative behavior towards coworkers^[[1]](#footnote-1)^ | **-1.676*** | 0.091 |

**p*<0.05, ***p*<0.01, *** <0.001 (two-sided tests)

Results from Table B thus indicate the existence of a selection bias in our sample. The next step we must take is to examine how this bias influences the way respondents interpreted the vignette characteristics. In other words, we must examine whether the effects of the independent variables differ with age, education, and self-reported cooperative behavior. Therefore, we ran Model 1 from Table 3 again including interaction variables between the independent variables and each of the variables on which we found the selection bias. Results can be found in Table C.

**Table C** Multivariate multilevel analyses to explain intentions to help while controlling for selection bias

|  | | | **Model 1**  Selection bias:  Age  (14,244 vignettes in 2,347 respondents in 127 organizations) | | **Model 2**  Selection bias:  Education  (14,280 vignettes in 2,380 respondents in 127 organizations) | | | | | | **Model 3**  Selection bias:  Coop. behavior  (14,268 vignettes in 2,378 respondents in 127 organizations) | |
| --- | --- | --- | --- | --- | --- | --- | --- | --- | --- | --- | --- | --- |
| **Fixed part** | | | Coef. | *S.E.* | Coef. | | | *S.E.* | | | Coef. | *S.E.* |
|  | | |  |  |  | | |  | | | | |
| Constant | | | 3.356*** | 0.094 | 3.379*** | | 0.095 | | | 3.345*** | | 0.094 |
|  | | |  |  |  | |  | | | | | |
| *Independent variables* | | |  |  |  | |  | |  | | |  |
|  | | |  |  |  | |  | | | | | |
| Training | | | 0.418*** | 0.024 | 0.420*** | | 0.025 | | 0.420*** | | | 0.024 |
| Skills and knowledge | | | 0.279** | 0.024 | 0.277*** | | 0.024 | | 0.278*** | | | 0.025 |
| Social norms | | | 0.448*** | 0.031 | 0.450*** | | 0.031 | | 0.453*** | | | 0.032 |
| Sanction | | | 0.112*** | 0.021 | 0.111*** | | 0.021 | | 0.114*** | | | 0.021 |
| Training*selection variable^1^ | | | -0.003 | 0.002 | 0.071*** | | 0.016 | | 0.003 | | | 0.032 |
| Skills and knowledge *selection variable | | | 0.001 | 0.002 | 0.032* | | 0.013 | | 0.026 | | | 0.038 |
| Social norms*selection variable | | | -0.006** | 0.002 | 0.039* | | 0.017 | | 0.031 | | | 0.039 |
| Sanction*selection variable | | | -0.001 | 0.002 | -0.009 | | 0.013 | | 0.144*** | | | 0.032 |
|  | | |  |  |  | |  | |  | | |  |
| *Control variables* | | |  |  |  | |  | | |  | |  |
|  | | |  |  |  | |  | | | | | |
| Sector (ref=Manufacturing) | | |  |  |  | |  | | |  | |  |
|  | Health care | | -0.079 | 0.118 | -0.108 | | 0.119 | | | -0.094 | | 0.118 |
|  | Higher education | | -0.106 | 0.101 | -0.224* | | 0.107 | | | -0.103 | | 0.102 |
|  | Transport | | -0.064 | 0.140 | -0.044 | | 0.138 | | | -0.054 | | 0.140 |
|  | Financial services | | 0.006 | 0.105 | -0.027 | | 0.106 | | | 0.027 | | 0.104 |
|  | Telecommunication | | -0.016 | 0.137 | -0.013 | | 0.133 | | | 0.033 | | 0.133 |
| Country (ref=Netherlands) | | |  |  |  | |  | | |  | |  |
|  | | Germany | 0.095 | 0.123 | 0.154 | | 0.126 | | | 0.108 | | 0.128 |
|  | | Portugal | 0.573*** | 0.090 | 0.584*** | | 0.088 | | | 0.569** | | 0.090 |
|  | | Bulgaria | 0.347*** | 0.089 | 0.340*** | | 0.085 | | | 0.353*** | | 0.084 |
|  | | |  |  |  | |  | | | | | |
| **Random part** | | |  |  |  | |  | | | | | |
| Variance (vignette level) | | | 1.180 | 0.046 | 1.180 | | 0.048 | | | | 1.185 | 0.016 |
| Variance (respondent level) | | | 1.133 | 0.074 | 1.322 | | 0.069 | | | | 1.303 | 0.075 |
| Variance (org. level) | | | 0.049 | 0.016 | 0.049 | | 0.015 | | | | 0.049 | 0.016 |
|  | | |  |  |  |  | | | | | | |
| **Fit statistics** | | | Parameter | DF | Parameter | DF | | | | Parameter | | DF |
| Deviance | | | 47702.012 | 20 | 47805.252 | 20 | | | | 47788.750 | | 20 |

**p*<0.05, ***p*<0.01, *** <0.001 (two-sided tests)

^1^ The selection variables are centered on their respective grand means

In Model 1 of Table C we explore whether the effects of training, skills and knowledge, and cohesiveness of the team differ depending on the age of respondents. Results show a significant negative effect for the interaction between the existence of social norms and age indicating that the positive effect of the existence of social norms on employees intentions to help coworkers is smaller for older employees than for younger employees. Given that older employees were more likely to participate in our experiment this implies that, if anything, the results we found are an underestimation of the actual effect. The other interaction effects are not significant.

In Model 2, we explore the selection bias caused by level of education. Results show that the effects of training, skills and knowledge, and the existence of social norms are stronger for higher educated respondents than for lower educated respondents. Given that the higher educated respondents were more likely to participate in the vignette experiment, this can be considered problematic. However, if we look at the effect sizes we see that the extra positive effect for being higher education is relatively small. For example, the positive effect of receiving training only increases with 0.071 points as the educational attainment of a respondent increases with 1 unit from the average educational attainment of respondents.

Results from Table B indicate that employees who scored high on the cooperative behavior scale in the survey were more likely to participate than employees scoring low on this scale. Model 3 explores whether this bias affects the independent variables. Results show that only the interaction effect with the existence of sanctioning mechanisms is significant. The positive effect of the existence of sanctioning mechanisms on employees intentions to help coworkers is stronger for employees who scored high on the cooperative behavior scale in the survey. This implies that the effect we find for the existence of sanctioning mechanism is partly due to this selection bias.

**Endnotes**

1. We constructed the variable measuring self-reported cooperative behavior towards coworkers as the mean over a scale with four items (alpha=0.759). The items in this scale are:

   - “I often help my colleagues solve work-related problems”
   - “I often help new colleagues adjust to the work environment”
   - “I often volunteer to cover work assignments for colleagues when needed”
   - “It is most important to me to communicate and work together well with my colleagues”

   Answer categories ranged from (1) “Strongly agree” to (5) “Strongly disagree”. The variable self-reported cooperative behavior was constructed as the mean over these four items (mean:4.080, std=0.559, range=1-5). The variable was recoded so that a higher score indicates more cooperative behavior. [↑](#footnote-ref-1)
